# Combining Transcriptome Assemblies from Multiple *De Novo* Assemblers in the Allo-Tetraploid Plant *Nicotiana benthamiana*

**DOI:** 10.1371/journal.pone.0091776

**Published:** 2014-03-10

**Authors:** Kenlee Nakasugi, Ross Crowhurst, Julia Bally, Peter Waterhouse

**Affiliations:** 1 School of Biological Sciences, University of Sydney, Sydney, Australia; 2 Mount Albert Research Centre, Plant & Food Research, Auckland, New Zealand; 3 The Centre for Tropical Crops and Biocommodities, Queensland University of Technology, Brisbane, Australia; The Ohio State University/OARDC, United States of America

## Abstract

**Background:**

*Nicotiana benthamiana* is an allo-tetraploid plant, which can be challenging for *de novo* transcriptome assemblies due to homeologous and duplicated gene copies. Transcripts generated from such genes can be distinct yet highly similar in sequence, with markedly differing expression levels. This can lead to unassembled, partially assembled or mis-assembled contigs. Due to the different properties of *de novo* assemblers, no one assembler with any one given parameter space can re-assemble all possible transcripts from a transcriptome.

**Results:**

In an effort to maximise the diversity and completeness of *de novo* assembled transcripts, we utilised four *de novo* transcriptome assemblers, TransAbyss, Trinity, SOAPdenovo-Trans, and Oases, using a range of k-mer sizes and different input RNA-seq read counts. We complemented the parameter space biologically by using RNA from 10 plant tissues. We then combined the output of all assemblies into a large super-set of sequences. Using a method from the EvidentialGene pipeline, the combined assembly was reduced from 9.9 million *de novo* assembled transcripts to about 235,000 of which about 50,000 were classified as primary. Metrics such as average bit-scores, feature response curves and the ability to distinguish paralogous or homeologous transcripts, indicated that the EvidentialGene processed assembly was of high quality. Of 35 RNA silencing gene transcripts, 34 were identified as assembled to full length, whereas in a previous assembly using only one assembler, 9 of these were partially assembled.

**Conclusions:**

To achieve a high quality transcriptome, it is advantageous to implement and combine the output from as many different *de novo* assemblers as possible. We have in essence taking the ‘best’ output from each assembler while minimising sequence redundancy. We have also shown that simultaneous assessment of a variety of metrics, not just focused on contig length, is necessary to gauge the quality of assemblies.

## Introduction

The challenges in assembling plant transcriptomes due to their inherent polyploidy has been highlighted in reports aiming to maximise the number of full length transcripts, minimising mis-assemblies, and distinguishing between homeologous transcripts [1]–[5]. The general consensus from these studies is that in order to reconstruct a broad range of sequences from the original transcriptome (transcript diversity), a wide number of parameters should be invoked in the initial assembly phase, in particular the k-mer size. This is not just applicable to plant transcriptomes, but also for a range of organisms [6]–[10].

It is also evident that even when similar parameters are applied across different assemblers, the output set of reconstructed sequences can be quite different [1], [2], [6], [8]. Similarly, given assembler-specific optimal parameters, different assemblers can be more efficient at reconstructing different sets of sequences [5], [6]. The TransAbyss assembler appears to perform better on lowly expressed genes, the Trinity assembler better on highly expressed ones, and the Oases assembler generally performs well on a large range of expression levels [6], [8]. However, while TransAbyss was able to reconstruct more authentic wheat transcripts than Trinity in one analysis [2], the reverse was true in another when using different parameters [11].

A further challenge in the assembly of polyploid transcriptomes is the ability to correctly reconstruct and distinguish between highly similar transcripts expressed from homeologous and paralogous genes of the sub-genomes. This becomes even more complicated with the presence of transcript isoforms. Oases has been reported to assemble sequences from deep sequencing data of hexaploid wheat that are up to 80% chimeric, and Velvet, Abyss (the genome assemblers required for Oases and TransAbyss), and Trinity performed only slightly better [12]. A high incidence of chimeric assemblies by these *de novo* assemblers has also been observed in human and mouse models [8].

Using only one assembler, TransAbyss, we have previously identified 33 transcripts representing 31 RNA silencing genes of the allo-tetraploid plant *Nicotiana benthamiana* [13] but 9 of them were only partially assembled, and had to be manually curated to generate the full length coding sequences. Given the many considerations regarding *de novo* transcriptome assembly described above, we wanted to generate a more complete *N. benthamiana* transcriptome by maximising the *de novo* assembled transcript diversity and completeness of assembled sequences. To achieve this we combined the output from four popular *de novo* transcriptome assemblers using a range of k-mer sizes and two datasets with differing read depths, into a super-set of sequences. This parameter space was also complemented biologically with data generated previously from 9 tissues of the plant, in addition to new data generated from the whole plant itself, increasing the chances of capturing any transcripts that are lowly or tissue-specifically expressed. Two pipelines, TGI clustering tools [14] (http://sourceforge.net/projects/tgicl/) and the EvidentialGene tr2aacds pipeline [15] (http://arthropods.eugenes.org/EvidentialGene/about/EvidentialGene_trassembly _pipe.html), were then implemented to reduce the redundancy acquired from pooling the assemblies. A variety of metrics, not just length-based ones, were used to assess all assemblies. We show that this multiple assembly combination approach followed by processing by the EvidentialGene tr2aacds pipeline maximises the diversity of *de novo* assembled transcripts and their completeness, while limiting sequence redundancy.

## Materials and Methods

### RNA isolation and deep sequencing

*Nicotiana benthamiana* plants were grown at 21°C under a 16-h photo-period and an 8-h dark period in an environmentally controlled glasshouse. Whole plants (6-week old) were washed and grounded in liquid nitrogen before storage at −80°C until further RNA extraction. Total RNA was isolated from 500 mg of the grounded tissues using the CTAB RNA extraction method [16]. Briefly, 5 ml of preheated (65°C) total RNA extraction buffer (2% (w/v) CTAB (Sigma), 2% (w/v) polyvinylpyrrolidone (PVP-40) (Sigma), 100 mM Tris HCl (pH 8.0), 25 mM EDTA (pH 8.0), 2 M NaCl and 2% β-mercaptoethanol) was added to each sample grounded in liquid nitrogen. Each sample was then extracted twice with an equal volume of Chloroform: Isoamylalcohol (24∶1), mixed and centrifuged at 4560 g for 20 minutes at room temperature. The resulting supernatant was carefully transferred into a new tube, mixed with LiCl at a final concentration of 2 M and incubated overnight at 4°C. After the incubation the samples were centrifuged at 4000 g for 20 min at 4°C and the resulting pellets were dissolved in 500 µl of preheated SSTE buffer (1 M NaCl, 0.5% SDS, 10 mM Tris HCl (pH 8.0), and 1 mM EDTA (pH 8.0)), extracted again with an equal volume of Chloroform: Isoamylalcohol and then washed with 75% ethanol and vacuum dried. Dried RNA pellets were diluted in RNAse free distilled water and stored at −80°C until used. The integrity of total RNA was determined by running samples on 1% denaturing agarose gel. The concentration and quality was initially assessed using a spectrophotometer (NanoDrop, Technologies Inc.) at an absorbance ratio of A260/230 and A260/280 nm. A Bioanalyzer (Agilent) was used to perform a final assessment on the quality prior to deep sequencing.

For whole plant samples generated for this study, RNA-seq library preparation and deep sequencing on Illumina HiSeq2000 instruments were carried out at the Australian Genome Research Facility and according to manufacturer's instructions. The Illumina TruSeq RNA sample preparation protocol was used to prepare libraries. The protocol results in fragment insert sizes ranging from 120 to 200 bp with a median size of 150 bp. Using these libraries, one round of 50 nt single-end sequencing run and two rounds of 100 nt paired-end sequencing runs were carried out. Libraries from the 9 tissue samples generated from our previous report [13] also utilised the Illumina TruSeq RNA sample preparation protocol with similar insert sizes. All raw reads generated from this study and previously generated reads have been deposited in the Short Read Archive (SRA) of NCBI under accession number SRA066161.

### Preprocessing of raw reads

Raw reads generated from this study, as well as reads previously generated from 9 tissue samples [13] were used for assemblies. Reads from all samples were pre-processed with the Trimmomatic software [17]. The first twelve bases of each read were trimmed, and bases at the 3′-end of each read that fell below a quality score of 20 were also trimmed off. A minimum length of 70 nt after trimming was applied. Orphaned reads were assigned as single-end reads. Processed reads from all samples were pooled into two datasets as properly paired reads and single end reads. Dataset 1 contained reads from the 9 tissue samples obtained previously and re-processed here, reads from the single-end sequencing run, and reads from the first round of paired-end sequencing. Dataset 2 contained all reads from dataset 1, and also the second round of paired-end sequencing.

All reads were formatted according to the specifications required by each *de novo* assembler.

### De novo transcriptome assemblies

Four *de novo* transcriptome assemblers were used: Abyss v1.3.4/TransAbyss v1.4.4 (Ta), Trinity r2013-02-25 (Tr), SOAPdenovo-Trans v1.01 (So), and Velvet v1.2.08/Oases v0.2.08 (Oa) [8]-[10], [18]–[20]. Assemblies were carried out using dataset 1 and dataset 2. For dataset 1, Ta was used to assemble k-mer sizes of 48 to 86 with a step size of 2, Tr was used to assemble a k-mer size of 25, and So was used to assemble a k-mer size of 31. For dataset 2, Ta was used to assemble k-mer sizes of 20 to 44 with a step size of 4 and k-mer sizes of 48 to 86 with a step size of 2, Tr was used to assemble a k-mer size of 25, So was used to assemble k-mer sizes of 21 to 81 with step size of 10, and Oa was used to assemble k-mer sizes of 25 to 75 with step size of 10.

K-mer assemblies from each assembler were also merged. For Ta and Oa, the native merge utilities in each software package were used. For Oa, we found that velvet contigs (*de novo* assembled transcripts) were represented as scaffolds, containing strings of N's connecting separate contigs. These were present despite setting the ‘no-scaffold’ option during assembly. Therefore prior to merging, scaffolds were split into contigs by removing the N's. For So, sequences from all k-mer assemblies were pooled and run through the TGI clustering pipeline (described below) to generate a merged assembly. Trinity only implements one k-mer size, and so no merging was performed. Contigs shorter than 200 nt were discarded from all assemblies.

Transcriptome assemblies were performed at two high performance computing facilities: the ‘Orange’ server provided by Intersect Australia Ltd (http://www.intersect.org.au), and the ‘Barrine’ cluster at the University of Queensland Research Computing Centre (http://www.rcc.uq.edu.au/).

### Reducing redundancy of assemblies

In order to reduce the redundancy of assemblies, ‘raw’ assemblies were first processed by CD-HIT-EST [21] with 100% identity to remove identical fragments. The processed sequences were then subjected to two processes: the TGI clustering tool [14] and the EvidentialGene tr2aacds pipeline (http://arthropods.eugenes.org/EvidentialGene/about/EvidentialGene_trassembly_pipe.html).

The TGI clustering tool (herein termed Tgi) is a pipeline that takes large numbers of EST and mRNA sequences which are first clustered based on pair-wise sequence similarity, after which each cluster is assembled by the CAP3 program [22] to produce longer, more complete consensus sequences. The following options were set for Tgi: “-p 99 -l 50 -v 100”.

The tr2aacds pipeline from the EvidentialGene package (herein termed Evi) selects a ‘best’ set of *de novo* assembled transcripts, based on coding potential, from a pool of such sequences. The algorithm first produces CDS and amino acid sequences for each sequence, and then removes redundant sequences using the amino acid information for choosing the best coding sequences from amongst identical sequences. Self-on-self BLAST is then implemented to identify highly similar sequences. The alignment data and CDS/protein identities are then used to select and output transcripts classified as ‘main’ (primary) or ‘alternate’, and another set classified as ‘dropped’ which did not pass the internal filters. The primary and alternate *de novo* assembled transcripts were used for further assessments.

### Mapping reads to each assembly

Two aligners were used to map reads to the assemblies: BWA v0.7.5a [23] and Bowtie2 v2.1.0 [24]. For BWA, the ‘bwtsw’ algorithm was used to index the database, and the ‘mem’ algorithm used to align the reads. The ‘sensitive’ option was utilised in Bowtie2. Samtools v0.1.18 [25], was used to interrogate the output .bam files for calculating mapping statistics.

### Database comparisons

The *de novo* assemblies were compared against five databases: the *N. benthamiana* v1 unigene build from Solgenomics (http://solgenomics.net/), the *N. benthamiana* v0.4.4 transcriptome and predicted protein databases from Solgenomics, the UniProtKB reference proteome set of *Arabidopsis thaliana* (April 2013), and the UniProtKB reference proteome set of *Solanum lycopersicum* (April 2013) [26]. Standalone NCBI-blast 2.2.26+ [27] was used for BLAST searches, with E-values of 1e-5 unless otherwise stated.

For querying RNA silencing gene transcripts (from [13]) against the *de novo* assemblies, BLASTn was used, with E-values of at least 1e-5 and considering only the top match, unless otherwise stated. A modified ‘analyze_blastPlus_topHit_coverage’ script from the Trinity software package (http://trinityrnaseq.sourceforge.net/) was used to calculate query and target database alignment coverage to determine whether the queries were assembled to full-length in each *de novo* assembly.

### Feature response curves (FRCs)

The FRC method [28], [29] implemented in this study is able to use read alignments from .bam files generated by various read mappers, including BWA and Bowtie2. The .bam files generated by BWA for calculating mapping statistics were used for the generation of FRCs. The FRC output text files were input into R v3.0 [30], and the ggplots2 library [31] was used to plot the curves.

### Assessment of mis-assemblies

Assemblies were compared against the v1 unigene dataset and v0.4.4 transcriptome assembly from Solgenomics using BLASTn, reporting a maximum of up to 3 matches using the ‘-max_target_seqs’ option and an E-value of 1e-5. Matches that contained only a single High-scoring Segment Pair (HSP) were identified using the Bio::SearchIO::Writer::TextResultWriter module from BioPerl [32], and from these, the ratio of the alignment length to the length of the query, and the ratio of the alignment length to the length of the subject, were calculated. Database matches giving less than 80% for both of these metrics were considered as potential mis-assemblies. A cut-off of 80% was chosen because of the presence of potential non-CDS and UTR sequences in the *de novo* assembled transcripts and reference databases.

### Annotation of the final transcriptome assembly

The assembly selected to be the ‘best’ representation of the *N. benthamiana* transcriptome was annotated by the in-house annotation pipeline, Bioview, as previously described [13]. Briefly, the databases used for annotation of the *de novo* assembled transcripts were SwissProt [33], UniRef90 [34] (http://www.uniprot.org/downloads), Plant RefSeq [35] and *Arabidopsis thaliana* proteins from TAIR10 (http://www.arabidopsis.org) using BLASTx v2.2.25 [27]. Annotations were based on matches first to SwissProt, then UniRef90, followed by matches to Plant RefSeq and then TAIR10. Annotations were then supplemented with descriptions such as “putative”, “probable” and “similar to”, based on ratios of HSP to query/subject lengths [13]. The annotated transcriptome assembly is available for interrogation and download at www.benthgenome.com.

### Other software

To interrogate, summarise and visualise the *de novo* assembled transcripts, BLAST results and read mappings, the following software were used: Circos [36], Geneious (http://www.geneious.com/), Integrative Genome Viewer [37].

## Results and Discussion

### Overview of assemblies

In our efforts to update our *N. benthamiana* transcriptome assembly, we initially generated a limited set of assemblies with dataset 1 (ds1) reads (see methods), which included previously generated sequencing data [13], and new reads generated from whole plant tissues (Table 1). Analysing a key set of RNA silencing gene transcripts showed that some sequences were more completely assembled when using Ta and others when using Tr (see case study for Dcl1, and Table S1). Also, incorporating additional sequencing data into ds1 (dataset 2 (ds2)), affected the completeness of the *de novo* assembled transcripts, even when using the same software (Table S1). The choice of assembler and variation in gene expression levels causing such alterations has been previously observed [1], [6], [8]. In short, it was not possible to reconstruct all of the RNA silencing gene transcripts to full-length using just one set of assembly conditions.

**Table 1:** Overview of assemblies generated from two datasets, showing k-mer size ranges and respective assemblies used to generate the two combined assembly types (SasmM and SasmK).

	Paired end reads	Single end reads	
**Dataset 1**	189,333,894	48,827,381	
**Dataset 2**	228,279,832	50,249,303	
	**A**	**B**	
	**Dataset 1 (ds1)**	**Dataset 2 (ds2)**	
**TransAbyss (Ta)**	k48-86, step size 2	k20-44, step size 4	**1**
**TransAbyss (Ta)**	-	k48-86, step size 2	**2**
**Trinity (Tr)**	k25	k25	**3**
**SOAPdenovo-Trans (So)**	k31	k21-81, step size 10	**4**
**Oases (Oa)**	-	k25-75, step size 10	**5**
**Combined assemblies**	**Contains:**		
SasmM (sum of merged assemblies)*	A1, A3, A4, B1+B2, B2, B3, B4, B5		
SasmK (sum of all k-mer assemblies)	A1, A3, A4, B1, B2, B3, B4, B4		

* k-mer assemblies merged by: Ta – Ta merge utility; So – TGI clustering software; Oa – Oa merge utility.

Following recommendations from the Evi pipeline [15], in order to reconstruct a transcriptome that recovered as many full-length *de novo* assembled transcripts as possible, four *de novo* transcriptome assemblers implementing a range of k-mer sizes and different input read depths were used to generate many sequences which were then pooled into a super-set. In total, 22 assemblies were generated from ds1 (427,495,169 reads) and 40 assemblies were generated from ds2 (506,808,967 reads) (Table 1). For the Ta, So and Oa assemblers, transcript merging utilities were used to merge the individual k-mer assemblies generated from ds2 reads, and a merged assembly was also generated separately from ds1 reads with Ta. Two approaches were then used to generate ‘combined’ assemblies. In the first approach, Tr assemblies and the merged assemblies were combined to generate a ‘Sum of merged assemblies (SasmM)’. In the second approach, all 62 individual k-mer assemblies using ds1 and ds2 reads from all assemblers were pooled into one large sequence set to generate a ‘Sum of k-mer assemblies (SasmK)’. A summary of these approaches is shown in Table 1.

The generation of multiple k-mer assemblies for each assembler and subsequent combination enabled the recovery of many potential transcripts and their homeologous variants, but resulted in very large assemblies containing many redundant sequences. Both Ta and Oa assemblies contained over 750,000 *de novo* assembled transcripts even after implementing their merging utilities, and the raw SasmM and SasmK assemblies contained 3.5 million and 10 million *de novo* assembled transcripts respectively (Table 2).

**Table 2:** Statistics of raw unprocessed k-mer assemblies (Tr and So) or merged k-mer assemblies (TaM and OaM), and their Tgi processed and Evi processed assemblies.

	TaMraw	TaMtgi	TaMevi	Trraw	Trtgi	Trevi	Soraw	Sotgi	Soevi
**Number of transcripts**	750713	178700	128938	284583	247174	44726	1248430	243483	95212
**N50**	906	1463	1231	1853	1809	1951	468	883	686
**Mean transcript length**	706	925	933	1017	966	1323	433	642	618
**Longest transcript**	14633	19508	14453	16069	16069	16069	15107	19151	15107
**Average length of top1000 longest proteins**	1778	1433	1405	1678	1552	1325	1239	1119	932
**% of complete CEGMA proteins**	97.18	99.6	93.55	97.58	97.58	90.32	49.19	87.1	47.18
**% of partial CEGMA proteins**	99.6	100	96.37	99.19	99.19	92.74	83.06	98.79	80.65
**Average bitscore to top 1000 longest Uniprot tomato proteins**	290.13	294.64	303.54	346.19	329.90	386.05	145.81	199.58	202.17
**Average bitscore to top 1000 longest Solgenomics ** ***N. benthamiana*** ** proteins**	319.99	344.99	333.77	420.45	403.25	434.89	163.87	240.74	224.79
**Mapping % of input reads(BWA)**	99.53	99.55	88.54	99.21	99.19	84.16	99.62	99.64	81.89
**Mapping % of chimeric reads mapQ>30 (BWA)**	0.081	0.265	0.237	0.480	0.536	0.381	0.062	0.570	0.344
**Mapping % of input reads (Bowtie2)**	97.54	97.17	79.93	91.53	91.40	74.01	96.80	96.38	71.18

	OaMraw	OaMtgi	OaMevi	SasmM raw	SasmM tgi	SasmM evi	SasmK raw	SasmK tgi	SasmK evi
**Number of transcripts**	797243	393516	72637	3562114	1066952	216177	9891564	735375	234526
**N50**	2024	2226	2131	1400	2079	1806	971	2162	2208
**Mean transcript length**	1407	1605	1671	910	1327	1283	681	1340	1674
**Longest transcript**	22494	22626	16312	22494	22494	16312	18177	20330	16038
**Average length of top1000 longest proteins**	2409	2033	1660	2811	2521	1840	3261	2456	2137
**% of complete CEGMA proteins**	99.19	99.19	90.73	99.19	99.6	97.18	99.6	99.6	98.79
**% of partial CEGMA proteins**	100	100	92.34	100	100	97.98	100	100	99.6
**Average bitscore to top 1000 longest Uniprot tomato proteins**	380.15	358.97	437.37	320.95	362.51	368.25	263.83	374.40	493.05
**Average bitscore to top 1000 longest Solgenomics ** ***N. benthamiana*** ** proteins**	442.55	423.07	503.58	365.16	421.91	418.70	298.63	449.56	554.61
**Mapping % of input reads(BWA)**	99.63	99.64	85.88	99.77	99.77	93.57	99.78	99.77	93.87
**Mapping % of chimeric reads mapQ>30 (BWA)**	0.015	0.033	0.450	0.002	0.013	0.124	0.002	0.016	0.179
**Mapping % of input reads (Bowtie2)**	94.73	94.55	72.40	98.90	98.83	88.82	98.79	98.81	88.86

To reduce the *de novo* assembled transcript numbers in our assemblies, the Tgi and Evi pipelines were used. Other studies have utilised the Tgi pipeline or CAP3 and reported substantial reduction in *de novo* assembled transcript numbers [2], [5]. Tgi does this by clustering and assembling similar (some partial) sequences generated from different assembly conditions, thus regenerating the full-length sequence which may not have otherwise been possible in individual k-mer assemblies. However, for allopolyploid plant transcriptomes, using such ‘cluster and assemble’ approaches may generate chimeric sequences from highly similar yet distinct transcripts generated by duplicated and homeologous gene copies. The presence of transcript isoforms complicates the process even more. Others have highlighted the difficulties of distinguishing homeologous transcripts at the k-mer assembly level which can be circumvented to a certain extent by varying the k-mer range (and indirectly the effect of expression levels) or using specialised approaches [1]–[4] and therefore adding an additional layer of merging through approaches such as CAP3 potentially adds to the difficulties further.

The importance of correctly distinguishing homeologous/paralogous copies of gene transcripts is particularly important where they have complementing or even separate functions. For example, it appeared that the partner copies of *Ago1* and *Ago4* in *N. benthamiana* could retain the overall function of the gene if only one of the copies were rendered non-functional [38]. The paralogs *Ta*FT and *Ta*FT2 in wheat interact with different targets in flowering regulation, and *Ta*FT2 is regulated by *Ta*FT [39]. This functional divergence is also mirrored in rice orthologs [40]. In *N. tabacum*, sub-functionalization of homeologous transcripts may occur on a limited scale, based on differential expression of a small percentage of these transcripts in its transcriptome [41].

The basis behind the Evi pipeline, which forgoes merging highly similar transcripts, is by generating as many *de novo* assembled transcripts as possible from a broad range of assembly conditions in the first instance, pooling them into one super-set of sequences as is (i.e. without any modifications such as merging), and then selecting from this a ‘best’ set of putative transcripts based on CDS and protein length and hence focusing on the coding-potential rather than just the transcript length.

Six primary assemblies were subjected to the Tgi and Evi pipelines: TaM (merged k-mer assemblies using ds2 reads), Tr (only one k-mer size using ds2 reads), SoM (merged k-mer assemblies using ds2 reads), OaM (merged k-mer assemblies using ds2 reads), SasmM and SasmK. This led to a total of 18 assemblies on which assessment was carried out. The unprocessed assemblies are defined here as the ‘raw’ assemblies, and Tgi and Evi processed assemblies are tagged as such.

### Assessment of assemblies

#### 1. Statistics of assemblies

The reduction of *de novo* assembled transcript numbers for all assemblies was most pronounced with the application of the Evi pipeline. In the SasmK assembly for example, there was over a 40-fold reduction in *de novo* assembled transcript numbers by Evi, whereas Tgi reduced these numbers by 13-fold (Table 2). Both cases reflect the high redundancy generated when combining multiple assemblies. The longest assembled sequences were produced in Tgi assemblies due to further assembly of similar sequences by CAP3. While the average length of the top 1000 longest predicted proteins was highest in raw and Tgi assemblies, the metric is inflated because of the redundancy of sequences. For example, when the top 1000 longest proteins from each assembly was clustered based on an identity of 95%, the number of unique clusters remaining was highest in the Evi processed assemblies (Table 3). Amongst the Evi assemblies, the SasmK assembly produced the largest average length of the top 1000 longest predicted proteins, at 2137 aa (Table 2).

**Table 3:** Number of protein sequences after clustering of top 1000 longest proteins in each assembly, using CD-HIT with an identity of 95%.

	Ta	Tr	So	Oa	SasmM	SasmK
**Raw**	288	326	348	124	71	45
**Tgi**	720	434	994	251	118	152
**Evi (primary + alternate transcripts)**	716	829	984	571	395	208
**Evi (primary transcripts)**	992	988	1000	971	958	973

The number of conserved core eukaryotic proteins detected from the CEGMA analysis [42], [43] was highest in the individual raw and Tgi assemblies (TaM, Tr, SoM, OaM), while the Evi pipeline appeared to filter out some of these sequences. However, the combined assemblies (SasmM and SasmK) yielded metrics that were similar to the raw and Tgi assemblies. The average bit-scores from BLAST matches to the top 1000 longest proteins from the Solgenomics tomato and *N. benthamiana* databases indicated that while the Tgi pipeline could offer better length-based metrics, the quality of assemblies in terms of homology detection was higher in Evi assemblies (Table 2).

The raw and Tgi assemblies had higher read mapping percentages compared to the Evi assemblies (Table 2). Since the mapping percentages reflect both unique and multi mapping reads and the Evi pipeline focuses on selecting *de novo* assembled transcripts with high coding-potential, sequences that were removed by the Evi pipeline may represent unspliced or non-coding transcripts. In the combined assemblies, the percentage of chimeric reads in Evi assemblies was higher than the raw and Tgi assemblies. This was at least partially due to these reads originating from intron/exon junctions in unspliced transcripts, which were removed by the Evi pipeline (data not shown). Once such unspliced sequences were removed, these reads showed chimeric (split) alignments to correctly spliced sequences and other sequences still containing a small length of intron (the Evi pipeline also does keep sequences containing some 5′ or 3′ untranslated regions). The proportion of chimeric reads in the Trraw assembly implies a higher percentage of unspliced or chimerically assembled transcripts, compared to other raw assemblies. This may be due to Tr only implementing one short k-mer size, as shown by the correlation of short k-mer sizes and higher rate of mis-assemblies [8]. Zhao et al. (2011) reported that Tr produced slightly higher numbers of fused (chimeric) transcripts compared to other assemblers when used for the *Drosophila melanogaster* transcriptome, but generated the lowest numbers when used for the *Schizosaccharomyces pombe* transcriptome [6]. It has also been reported that Oa and Tr produce higher numbers of chimeras than Ta [5]. This again highlights the variability of generating transcriptome assemblies, which depends on the organism, assemblers and parameters used.

#### 2. Feature response curves

Feature response curves (FRCs) have been used to evaluate the trade-off between the contiguity and correctness of assemblies. This metric is based on the principle that the assembly correctness can be predicted by identifying on each *de novo* assembled transcript, ‘features’ representing potential errors or complications during the assembly process [28], [29]. Such features were developed for assessing genomes where the coverage is on average uniform, but several of these features can also be applied to transcriptomes (F. Vezzi, personal communication). The ‘High_Spanning_PE’ feature, is based on the number of reads for which each mate of a read pair is mapped onto two different *de novo* assembled transcripts. The coverage of the estimated transcriptome size is calculated from sequences where the total sum of these features are less than a threshold, and then plotted as a function of the threshold to generate FRCs. Assemblies with curves that show higher coverage at lower feature thresholds can be considered of higher quality.

**Figure 1:**
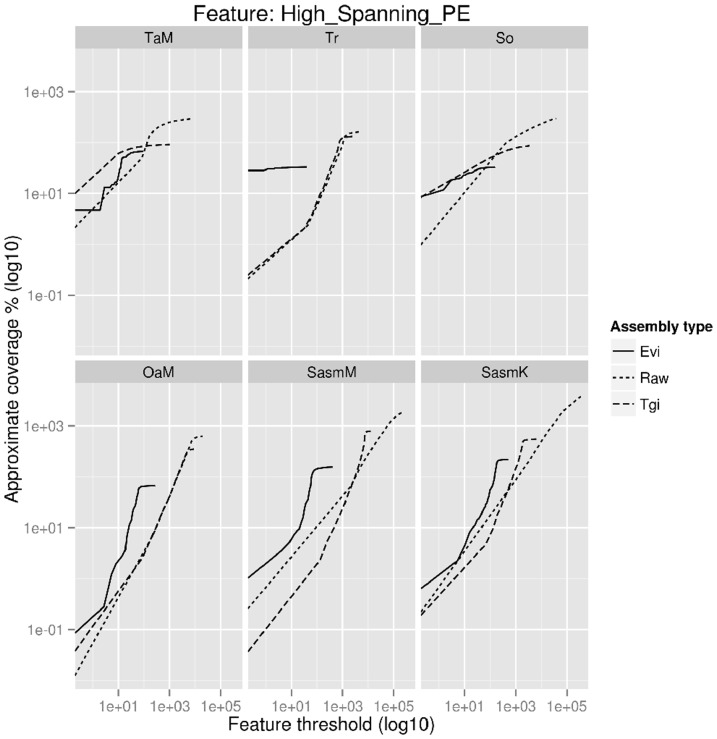
Feature response curves of assemblies using the ‘High_spanning_PE’ feature. This feature measures the number of PE reads where the pairs are mapped onto different contigs (*de novo* assembled transcripts). The feature threshold is used to filter out contigs that fall above a threshold. That is, only contigs that contain less than a threshold number of features are used to calculate the coverage at that threshold. Except for the TaMevi, the Evi processed assemblies appear to perform best or at least on par on all assemblies (raw vs Tgi vs Evi) as higher coverage is achieved at a lower feature threshold.

Figure 1 shows the outcome of FRCs with the ‘High_Spanning_PE’ feature of all 18 assemblies. With the exception of TaMevi, processing by the Evi pipeline appeared to retain more *de novo* assembled transcripts onto which paired reads could be concordantly mapped. Inspection of a sample of sequences that were designated as ‘High_Spanning_PE’ showed that these *de novo* assembled transcripts were either not fully assembled and hence paired reads could only map to the terminal regions of partially assembled sequences of the same transcript, or chimeric where alignment of the sequences to the genome verified that they were as such (data not shown). The higher redundancy of sequences in raw and Tgi assemblies most likely caused the slower increase of coverage at greater feature thresholds, and with that regard, FRCs could be used as a metric to assess the redundancy of transcriptome assemblies. The FRCs for the Tgi processed assemblies of SasmM and SasmK showed a lower increase in coverage compared to their raw assemblies in contrast to the other assemblies (Figure 1). This may also be an indication of higher numbers of chimeric sequences as a result of clustering and assembling very high numbers of similar sequences that were pooled in the combined assemblies. Indeed, this appears to be the case in subsequent analyses (see section 6). Based on these assessments of the FRCs, the Evi pipeline appeared to generate the highest quality assemblies.

#### 3. Comparison to reference databases

Comparison of the *de novo* assembled transcripts from each assembly to tomato and *A. thaliana* reference databases showed that the combined assemblies SasmM and SasmK yielded the highest number of matches for both total hits to the database, and for transcripts that had more than 80% alignment coverage of the target (Table S2). The Oases assembler also performed well compared to the other assemblers when assessed separately. There was in general not a large difference in database matches between the Tgi and Evi assemblies, and the number of database matches generated from the SasmM and SasmK assemblies were comparable.

**Figure 2:**
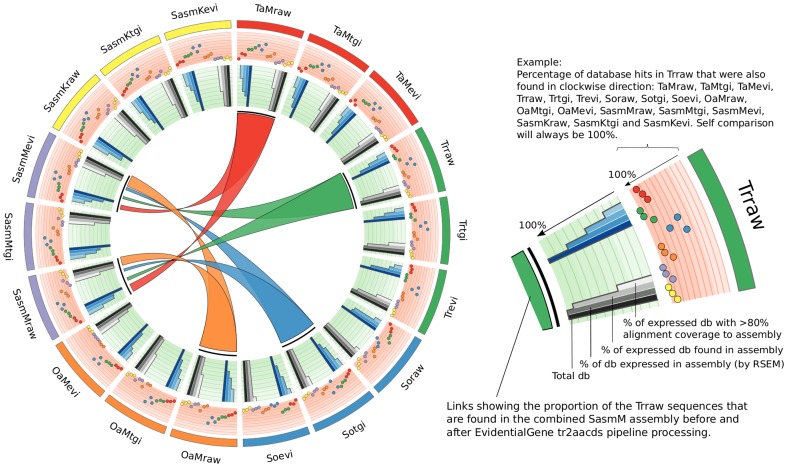
Summary of BLASTx comparisons to the Solgenomics *N. benthamiana* v0.4.4 predicted proteins database. The orange tracks show the percentage of database hits from each assembly that were also found in other assemblies, represented by 18 data points in each track, including self-on-self comparison (see legend). The green tracks show the percentage of the database that were found in each assembly. The grey histograms are comparisons to the top 1000 longest proteins in the database, and the blue histograms are comparisons to all sequences in the database. Each set of histograms are sub-divided into 4 bars, representing from darkest to lightest colour, the total database (always 100%), the percentage of the database that is actually expressed in our assembly, the percentage of these expressed database sequences that were found in the assembly, and of these the percentage that were aligned to more than 80% of the database sequence length. The coloured links show the proportion of TaMraw, Trraw, Sotgi and OaMraw *de novo* assembled transcripts that were present in the SasmMraw and SasmMevi assemblies.

To also assess our assemblies against an independent *N. benthamiana* database, our sequences were compared to a predicted protein set from Solgenomics. This database (v0.4.4) has 76,379 predicted proteins, and of these, 57,411 of the corresponding transcript sequences were deemed to be expressed based on RSEM analysis [44] using our RNA-seq reads. The percentage of matches reported here are with respect to the proteins deemed to be expressed in our samples. Figure 2 (green track) summarizes the proportion of Solgenomics *N. benthamiana* proteins (total and top 1000 longest proteins) detected by our assemblies. Most of the assemblies were able to reconstruct more than 90% of the top 1000 longest, expressed proteins (Figure 2, third bar in grey histograms). However, of these, only the SasmM, SasmK and Oases assemblies could report more that 50% of these transcripts as being assembled to more than 80% of the target length. Comparing the assemblies against the entire Solgenomics *N. benthamiana* database proteins (Figure 2, blue histograms) showed more variable target alignment coverage, but again the SasmM and SasmK assemblies displayed the most number of matches against the database.

For each assembly, the proportion of sequences that aligned to 80% of the target database sequence length and that were also detected in other assemblies was assessed (Figure 2, orange tracks). For example, 63.5% of database matches from the Taraw assembly were also found in the Trraw assembly. These pair-wise comparisons can firstly be used assess the ability of an assembler to assemble more complete sequences, and secondly to assess how well the SasmM and SasmK assemblies retained sequences from each of the individual assemblies after Tgi and Evi processing. As an example, we will focus on a discussion of the TaMevi and Trevi assemblies. In the TaMevi section of Figure 2, the green dots in the orange track indicate that 68%, 68% and 63% of the database matches detected in the TaMevi assembly were also found in the Trraw, Trtgi and Trevi assemblies, respectively. This means that over 30% of the database matches in the TaMevi assembly were not detected in the Tr assemblies. Conversely, in the Trevi section of Figure 2, approximately 90% of its database matches could be also detected in the TaMraw, TaMtgi and TaMevi assemblies. Based on these pair-wise comparisons, it would appear that the Ta assembler is able to reconstruct a higher number of more completely assembled transcripts (i.e. >80% of target sequence length) when compared to the Tr assembler.

In fact, based on the approach used here, it appears that for the *N. benthamiana* transcriptome, the Ta assembler could generate the most number of transcripts assembled to >80% target sequence length amongst the four assemblers tested, closely followed by Oa, and then Tr and So. The pair-wise comparisons showed that the combined assemblies (SasmM and SasmK) processed by Tgi and Evi still appeared to retain much of the *de novo* assembled transcript diversity produced from each of the four individual assemblers, as reflected by the higher percentage of matches to the reference database in each individual assemblers' tracks, but lower percentages in the combined assemblies' tracks.

Figure 2 (coloured ribbons) also shows that the TaM and OaM assemblies contributed the most number of sequences to the raw SasmM assemblies (represented by the width of the coloured ribbons), but the Evi pipeline retained a larger proportion of OaM generated sequences. This was also the case for SasmK assemblies (data not shown).

#### 4. Detection of full-length RNA silencing gene transcripts

**Figure 3:**
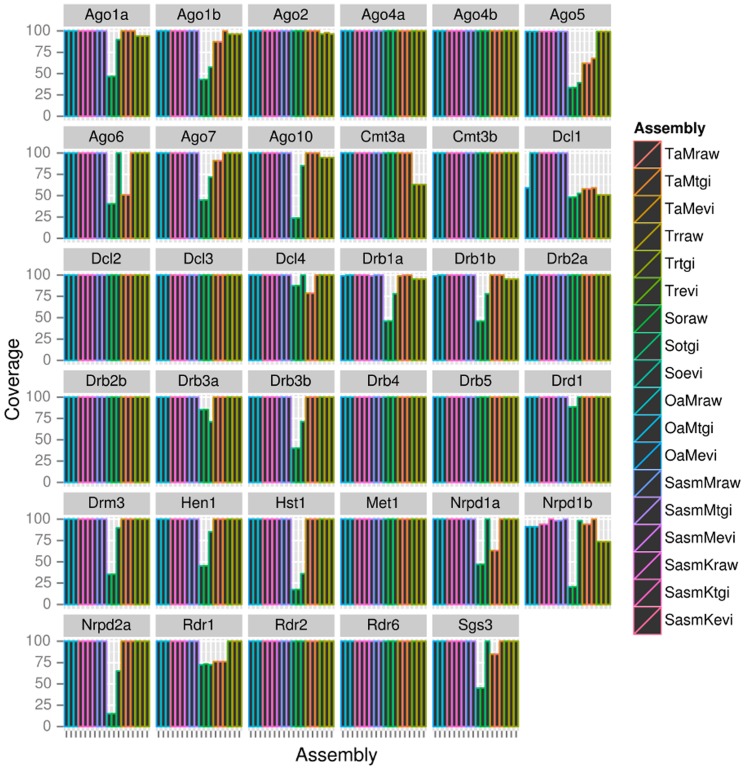
Alignment coverage of 35 RNA silencing gene transcript sequences. Sequences were queried against the 18 transcriptome assemblies in this study. The CDS of these *de novo* assembled transcripts were screened against the assemblies using BLASTn, and the query alignment coverages were calculated from the best match to the database.

One of the primary purposes of updating our *N. benthamiana* transcriptome assembly was to enable the detection of full-length RNAi silencing transcripts from first pass BLAST searches. By combining sequences from multiple assemblies, we were able to detect to more than 90% query alignment coverage, all 33 RNAi sequences described previously [13] in a single assembly. In the SasmKevi assembly, the Nrpd1b transcript had a 94% query coverage, Ago5 and Drd1 had >99% coverage, whereas all other query sequences had a 100% alignment coverage. The SasmKtgi assembly displayed 100% query alignment coverage for all transcripts except Ago5, which showed a 99.2% coverage. Two new variants of Drb1 and Drb3 (designated Drb1b and Drb3b) were also detected. From these results, there appears to be two transcript copies of Drb1, Drb2 and Drb3 in the *N. benthamiana* transcriptome. The alignment coverage of all query sequences to assemblies tested in this study, including newly identified and updated variants, is summarised in Figure 3.

In addition to the identification of Drb1b and Drb3b, several RNAi sequences were updated in length, including Ago4b, Ago5, Dcl3 and Drb4 (Table S3). The Dcl3 sequence was previously reported to lack the DEAD helicase motif in the N-terminus [13]. However, due to the additional data and new assembly methodology implemented in this study, the Dcl3 sequence has been supplemented with an additional 600 nt, which does contain a helicase motif. The updated RNAi sequences are available at our web portal, www.benthgenome.com.

#### 5. Detection of paralogous transcripts

The benefits of generating assemblies from multiple assemblers are not only reflected in full-length transcript assembly of our RNA silencing genes, but also in the detection of paralogous or homeologous copies of certain transcripts. Of the 35 silencing transcript sequences used in this study, six were determined to be of paralogous or homeologous origin, based on their nucleotide and amino acid identities to each other (Table 4), and homology to known RNA silencing genes in other organisms [13]. In this study, the Tr assembler only recovered one of the variants corresponding to Ago1, Drb1, Drb2 and Drb3, even after Tgi and Evi processing (Table 4). For the So assembler, only one variant of Ago1 could be recovered, and it was not able to distinguish between Drb3a, Drb3b and Drb5. When the So assemblies were subjected to the Tgi pipeline, only Drb2b could be detected, indicating a case where merging of sequences post-assembly may create chimeric sequences and hence reduce *de novo* assembled transcript diversity within assemblies. The Oa assembler could not distinguish between Drb1a and Drb1b, and only generated a sequence most similar to Drb1a. The Ta assembler was able to resolve all sequence variants. These observations coincide with another study that showed that the Oa and Tr assemblers do not perform well in distinguishing between highly similar paralogous or homeologous transcripts [12]. Although one could argue that percent identities should display a positive correlation with the ability to differentiate and assemble paralogous transcripts, this does not appear to be the case (Table 4), further supporting the case that there is no one ‘perfect’ condition for transcriptome assembly, especially where highly similar transcripts exist.

**Table 4:** Homeologous or paralogous RNA silencing gene transcript sequences, their nucleotide and protein identities, and whether they could be assembled separately in TaMraw, Trraw, Soraw and OaMraw assemblies.

				Homeologs/Paralogs distinguished?			
Homeologs/Paralogs		% nucleotide identity	% protein identity	TaMraw	Trraw	Soraw	OaMraw
Ago1a	Ago1b	81.40%	84.80%	Yes	No	No	Yes
Ago4a	Ago4b	82.90%	85.00%	Yes	Yes	Yes	Yes
Cmt3a	Cmt3b	70.60%	65.60%	Yes	Yes	Yes	Yes
Drb1a	Drb1b	96.10%	93.00%	Yes	No	Yes	No
Drb2a	Drb2b	94.80%	94.30%	Yes	No	Yes	Yes
Drb3a	Drb3b	95.40%	94.50%	Yes	No	No	Yes

#### 6. Proportion of mis-assemblies

The percentage of *de novo* mis-assembled transcripts was gauged by comparing the assemblies to the top 1000 longest sequences from the Solgenomics *N. benthamiana* v1 unigene dataset (NbUnigene), and the v0.4.4 transcriptome assembly (NbTrans) also hosted on that site. *De novo* assembled transcripts from our assemblies and reference database sequences that both did not align to more than 80% of their respective lengths (see Methods) were considered as potential mis-assemblies. This measure may also be affected by the presence of alternatively spliced transcripts, but nonetheless does provide an indication of the frequency of such sequences (mis-assembled or alternatively spliced) within the assemblies.

This comparison showed that the Tgi assemblies contained a higher number of mis-assemblies when compared to the Evi assemblies and even the raw assemblies. This is based on the higher percentage of single HSPs that were less than 80% of both the query and subject sequence lengths (Table 5), and is in spite of the reduction of *de novo* assembled transcript numbers (from the raw assemblies) by the Tgi pipeline. It was also clear that the Evi assemblies contained substantially more sequences that were aligned more completely when compared to these databases, with the exception of the SoMevi and NbUnigene comparison (Table 5). In combination with other metrics described thus far, the So assembler does not appear to perform well for the transcriptome assembly of *N. benthamiana*.

**Table 5:** Percentage of lone high-scoring segment pairs (HSPs) (i.e. from alignments where there was only one single HSP) from18 assemblies compared to two databases, where the HSP length was more or less than 80% of both the query and target database length.

Compared to NbUnigene*						
	<80%			> = 80%		
	Raw	Tgi	Evi	Raw	Tgi	Evi
TaM	50.2	73.1	26.5	4.6	9.8	13.6
Tr	56.2	53.5	33.1	14.5	16.5	30.7
So	48.5	71.2	26.3	1.0	3.5	3.3
OaM	69.6	79.0	53.5	11.6	8.0	24.3
SasmM	56.9	68.4	37.7	7.1	7.3	17.0
SasmK	53.2	78.2	45.1	5.8	7.1	26.5

Compared to NbTrans*						
	<80%			> = 80%		
	Raw	Tgi	Evi	Raw	Tgi	Evi
TaM	71.6	82.2	46.4	2.2	3.4	11.3
Tr	73.5	72.3	41.6	2.7	3.0	13.3
So	69.3	83.8	51.3	1.7	2.4	9.5
OaM	79.3	83.3	47.4	2.9	3.1	17.9
SasmM	75.1	81.1	51.7	2.6	1.9	10.3
SasmK	66.1	84.0	54.9	2.6	1.9	11.5

*NbUnigene: top 1000 longest sequences from the v1 Unigene build from Solgenomics. NbTrans: Total v0.4.4 transcriptome assembly from Solgenomics.

The relatively low proportion single HSPs that covered more than 80% of both query and subject lengths in the Evi assemblies is also affected by the incompleteness of the reference databases. For example, a 2 kb *de novo* assembled transcript may have aligned to a 1 kb NbUnigene sequence to generate a single HSP, resulting in a 50% alignment coverage for the query sequence, but a 100% alignment coverage for the NbUnigene sequence. This kind of alignment is not reflected in Table 5. In addition, details of the transcriptome assembly hosted on the Solgenomics database are unclear, and differences in tissue samples, read depth and assembly methods could account for the relatively low number of matches to our transcriptome (the highest percentage of matches to the NbTrans database was from the SasmKraw assembly at 76.6%). Nevertheless, these comparisons show that the Evi assemblies do appear to contain less numbers of potentially *de novo* mis-assembled transcripts, and that the Tgi pipeline appear to increase the numbers of mis-assemblies. Indeed, high numbers of chimeric sequences were reported when using CAP3 (a part of the Tgi pipeline) in Ta and Tr assemblies [1], and conversely that CAP3 does not serve to reduce mis-assemblies despite reducing redundancy [5].

### Selection of assembly for annotation

The SasmKevi assembly was selected as our updated *N. benthamiana* transcriptome based on the overall assessment of all metrics applied in this study. Although the SasmM combined assemblies also displayed good overall metrics, the average BLAST bit-scores and the average predicted protein lengths were not as high as the SasmKevi assembly (Table 2). In addition, the raw *de novo* assembled transcripts in TaM and OaM were initially subjected to a merging step prior to transcript pooling, introducing the possibility of mis-assemblies of sequences with high similarity, as shown in Table 5. The number of primary or main *de novo* assembled transcripts as classified by the Evi pipeline was 49,818, which is similar to the 44,000 to 53,000 *de novo* assembled transcripts identified in the *N. sylvestris* and *N. tomentosiformis* transcriptomes [45]. The number of alternate transcripts or possible spliceforms classified by the Evi pipeline was 184,708.

The sequences in our *de novo* assembly were annotated by the in-house annotation pipeline, Bioview, and were compared to four databases: SwissProt, Plant RefSeq, UniProt90 and *A. thaliana* TAIR 10 proteins. Table 6 compares these results to our previous assembly (Nbv3), and shows a marked improvement in the number of homologous proteins detected. Tissue specific read mapping percentages show a high alignment rate for SasmK, but our previous assembly still showed a higher read mapping percentage. This is probably due to the higher sequence redundancy of the previous assembly, and the presence of sequences (e.g. non-coding or isoforms) that were filtered out by the Evi pipeline.

**Table 6:** Comparison of SasmK (this study) and Nbv3 [13] (previous version) transcriptome assemblies against four protein databases, and tissue specific read mapping percentages (using bowtie2).

**Database matches**				
	**SwissProt**	**RefSeq Plant**	**UniProt**	**TAIR proteins**
**SasmK**				
Total matches to database	229650	231748	230545	233251
Unique matches (E-value <1e-3)	174780	213504	214644	203709
Percentage of transcriptome	74.5	91.03	91.52	86.86
**Nbv3**				
Total matches to database	204575	214605	208399	224225
Unique matches (E-value <1e-3)	122003	166440	166216	155596
Percentage of transcriptome	51.4	70.13	70.03	65.56
**Tissue specific mapping percentages**				
	**SasmK**	**Nbv3**		
Apex	85.46	92.66		
Capsule	89.32	94.99		
Drought stressed leaves	82.81	92.83		
Flower	82.51	95.56		
Leaf	86.99	95.9		
Roots	91.58	97.32		
Seedling	91.88	97.58		
Stem	87.86	94.77		
Tissue culture	88.93	95.21		
Whole plant	88.67	95.9		

### Case study: Dcl1

**Figure 4:**
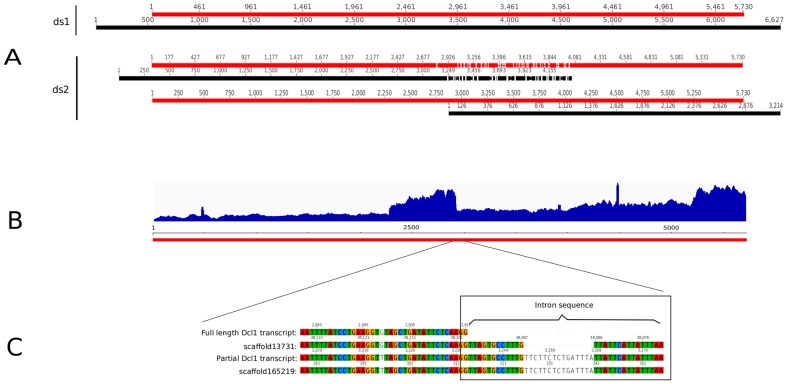
Dcl1 transcript assembly status by the Trinity assembler. Red bars indicate the known Dcl1 CDS used as query. Black bars indicate the transcripts assembled by the assembler. A: Alignment of *de novo* assembled transcripts generated with ds1 and ds2 reads to the query Dcl1 sequence. A full length Dcl1 sequence could be assembled with ds1 reads, but only two partial sequences were assembled with ds2 reads. B: Read depth profile from all RNA-seq reads mapped to the query Dcl1 CDS. Changes in read depth could indicate the presence of various isoforms. C: Alignment of the full length and partial Dcl1 *de novo* assembled transcripts at the region where there is a sharp change in read depth, to respective genome scaffolds in our v0.3 draft assembly. This shows that the partially assembled Dcl1 sequence contains unspliced intron, and that there may be two loci for Dcl1 as implied by the different intron sequences in the scaffolds (see text).

One of the query RNAi silencing transcripts, Dcl1, was assembled differently depending on the assembler and parameters invoked. Using ds1 reads, the 5.7 kb CDS of the transcript could be assembled as a single full-length sequence with the Tr assembler, but with ds2 reads, it could only generate two partially *de novo* assembled transcripts (Figure 4A). In contrast, the Ta assembler could only generate two partially *de novo* assembled transcripts with both ds1 and ds2 reads. The Oa assembler however could assemble a single full-length Dcl1 sequence even with ds2 reads (ds1 reads were not tested).

When the input RNA-seq reads were aligned to the Dcl1 CDS, it was clear that the region between 2,500 bp and 3,000 bp was elevated in read depth (Figure 4B). This region corresponded to the region where partially assembled Dcl1 sequences overlapped in the assemblies where it could not be fully assembled. Closer inspection of this region revealed that the partially assembled Dcl1 transcripts contained unspliced intron sequence which mapped to scaffold165219 in our v0.3 draft genome assembly, whereas the full length sequence mapped to scaffold13731 (Figure 4C). Scaffold13731 contained a different intron sequence in this region to scaffold165219. Although this could imply two Dcl1 loci, scaffold165219 is only 814 nt long, making it difficult to draw any strong conclusions due to the possibility of incomplete genome assembly. Nonetheless, reads containing this intron sequence were present in both ds1 and ds2 read pools, and implied that increased numbers of such reads in ds2 affected complete Dcl1 assembly in the Tr assembler (and also the Ta assembler). In comparison, such reads did not appear to affect complete Dcl1 assembly in the Oa assembler. A similar situation was observed with the ESM1 and rbcS transcripts in *Pachycladon fastigiatum*, an allopolyploid plant, where higher number of reads caused increasingly fragmented assemblies of these sequences [1].

In *A. thaliana*, the levels of Dcl1 mRNA transcripts are self-regulated by an intronic miRNA, which is derived from a hairpin that is formed within intron 14 of the Dcl1 transcript [46]. When enough miRNA is produced from this intron sequence, the mRNA is cleaved in the vicinity of this intron, resulting in two fragments of approximately 4 kb and 2.5 kb. These are comparable in size to our partially assembled *N. benthamiana* Dcl1 sequences (approximately 4.1 kb and 3.2 kb). A similar mechanism may also be occurring in *N. benthamiana*, although we could not detect such a miRNA from our small RNA sequence libraries (data not shown). The miRNA responsible for Dcl1 regulation is present at very low levels in *A. thaliana* [46], [47].

## Conclusion

We have generated multiple assemblies from four *de novo* transcriptome assemblers, and combined their output into a super-set of *de novo* assembled transcripts. The assemblies were processed by the Tgi and Evi pipelines and assessed using a number of measures. Length-based metrics indicated that Tgi processed assemblies contained longer, and in some cases more completely assembled, *de novo* assembled transcripts than the original reference databases. However, other measures such as the average BLAST bit-scores of homology matches, feature response curves, the substantial reduction of number of redundant sequences, and proportion of potentially *de novo* mis-assembled transcripts, showed that the Evi pipeline generated higher quality assemblies. Moreover, while the TaMevi, Trevi, SoMevi and OaMevi assemblies mostly displayed lower length based metrics compared to the Tgi assemblies, the combined SasmKevi assembly substantially improved the entire spectrum of these metrics. This was well illustrated by the reconstruction of sequences from the paralogous copies of the Ago and Drb gene family members and the full length reconstruction of the Dcl1 transcript sequence. Although the approach requires high computational and storage capabilities, it generates more completely *de novo* assembled transcripts and a higher read mapping percentage than individual assemblies alone, and may be particularly useful for many of the polyploid crop species used in agriculture.

The need to use a ‘wide-spectrum’ approach described here depends on the biological aim of constructing a transcriptome assembly. In *N. tabacum*, it appears that there is no preferential expression of genes from either of its sub-genomes despite a relatively high percentage of homeologous genes, and hence for functional analysis purposes, it may not always be necessary to attempt to distinguish homeologous transcripts [41]. On the other hand, there are also methods that attempt to de-convolute paralogous/homeologous chimeric sequences after the initial transcriptome assembly [3], [4].

The latest *N. benthamiana* transcriptome assembly, containing both primary and alternate spliceforms as classified by the EvidentialGene tr2aacds pipeline, is available for download and interrogation through BLAST and Gbrowse at www.benthgenome.com.

## Supporting Information

Table S1 Alignment coverage of query RNA silencing gene transcript sequences to TransAbyss and Trinity assemblies, using dataset 1 and dataset 2 reads.
(XLS)

Table S2 Number of matches found in each of 18 assemblies when compared to the UniProtKB database sequences of tomato (*Solanum lycopersicum*) and *Arabiodopsis thaliana* (compared to the top 1000 longest and total database proteins).
(XLS)

Table S3 Summary of updated CDS and protein lengths of RNA silencing gene transcript sequences in *N. benthamiana*.
(XLS)
